# The use of spiral CT in the detection and management of a permanent maxillary first molar with single root and single canal: A rare occurrence

**DOI:** 10.4317/jced.54074

**Published:** 2017-09-01

**Authors:** Neil De Souza, Krishna Shetty, Rohit P. Kolipaka, Paul Chalakkal

**Affiliations:** 1 Lecturer, Department of Pedodontics and Preventive Dentistry, Government Dental College and Hospital Bambolim, Goa

## Abstract

A sound knowledge of the normal tooth morphology and its numerous variations is essential to the success of endodontic therapy. While extra canals and lateral canals are routinely encountered and documented, there also exists the rare possibility of a fewer number of canals than normal. Early detection of such an occurrence is critical to the prevention of iatrogenic errors during endodontic treatment. The advent of spiral CT as a diagnostic tool serves as a much needed confirmation to understand the complex three dimensional anatomy of teeth. The aim of this case report is to highlight one such case reporting unusual morphology of the permanent maxillary first molar displaying single canal and single root, and the role of spiral CT in its detection and management.

** Key words:**Permanent maxillary first molar, root canal anatomy, single root, single canal, Spiral CT.

## Introduction

An adequate knowledge of the root canal anatomy and its variations is vital to the clinician’s goal of obtaining a technically satisfactory endodontic outcome. Adequate access, vision, biomechanical preparation and obturation are the foundation to long term endodontic success. Variations in dental anatomy and canal morphology are found in all teeth, especially in multi rooted teeth. The presence of extra canals, lateral canals and apical ramifications are commonly encountered and well documented (1,2). The variations in the root canal morphology of the maxillary 1st molars is particularly common, especially the presence of an extra mesiobuccal canal. Case reports of the occurrence of 4, 5 and 6 roots with a corresponding number of canals have been reported in past literature. However, the configuration of a single canal in a single rooted maxillary first molar has rarely been described, that to in a pediatric age group.

Conventional intra-oral radiography serves as an important diagnostic tool in Endodontics. However its limitations are well documented. Recently, newer diagnostic methods such as computed tomography (CT) and Spiral (SCT) or Helical CT have emerged as powerful tools for evaluation of root canal morphology. This case report presents a maxillary first molar with an unusual morphology of a single root and a single canal, and highlights the importance of SCT as a diagnostic tool to confirm the same.

## Case Report

A 10-year-old female patient whose medical history was noncontributory presented to the clinic with chief complaint of pain in upper right posterior region of jaw. She was having intermittent pain for past six months, which had aggravated since the past four days. Intraoral examination revealed that the right maxillary first molar was carious and tender on percussion. Thermal and electrical pulp testing elicited a negative response in maxillary left first molar. The preoperative radiograph shows widening of periodontal ligament space with right maxillary first molar. The radiograph also revealed an unusual anatomy of involved tooth with single root and single canal. Based on the clinical and radiographic evaluation, diagnosis of irreversible pulpitis with acute apical periodontitis of right maxillary first molar was made, and endodontic treatment was planned.

To ascertain the unusual morphology, multiple X-rays in variable horizontal angulations were taken. These X-rays revealed a broader single canal bucco-lingually. In order to confirm the apparent finding of the IOPA radiographs, the patient was referred for CBCT imaging of tooth 16 with 3D reconstruction. CBCT was performed for maxilla using dental software Dentascan. 3D image of the maxilla was obtained, the tooth in question was focused and its morphology was obtained in longitudinal and trans-verse cross section of 0.5 mm thickness. The results of the CBCT showed the presence of a single root with the Vertucci’s type I canal configuration. Thereafter, the root canal treatment was initiated.

**Figure 1:**
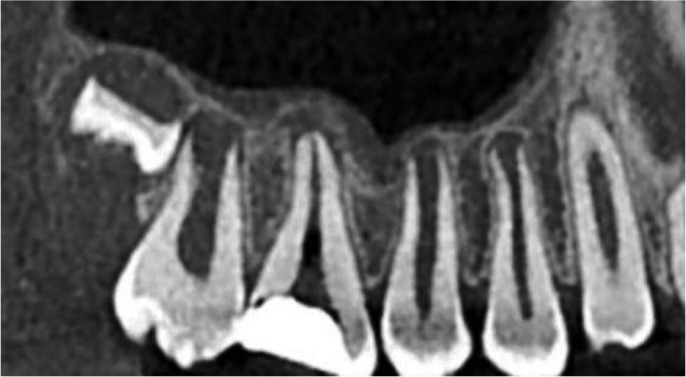
Preoperative radiographic view of maxillary first molar.

**Figure 2:**
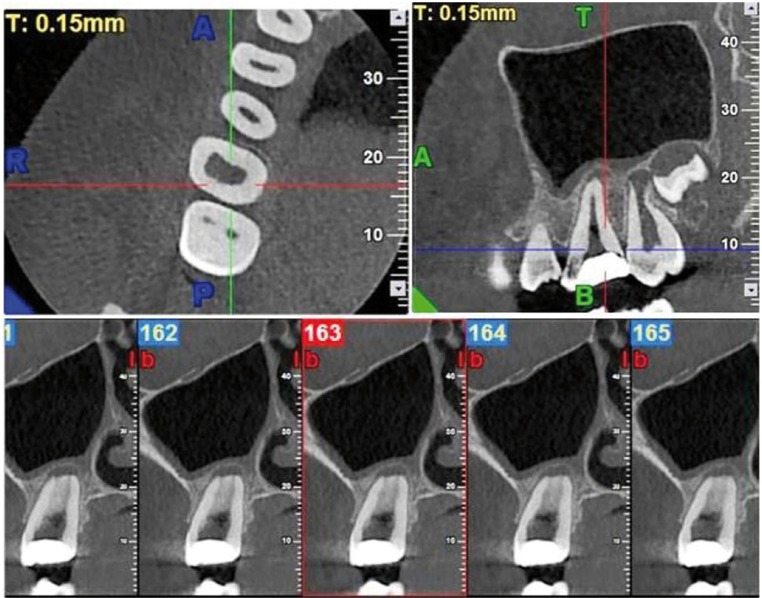
Preoperative occlusal, proximal and buccal CT view of maxillary first molar.

**Figure 3:**
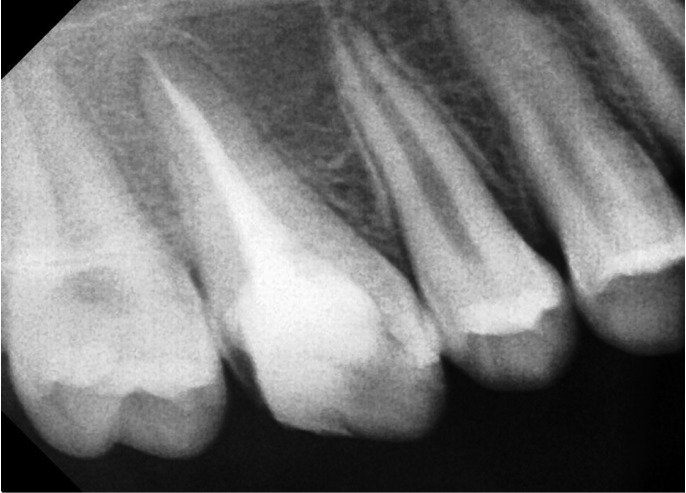
Radiographic view showing 2 year follow up of obturated maxillary first molar.

Access opening was done in right maxillary first molar. On examination, clinical presence of single wide orifice was found in the center of the pulpal floor. Further inspection of the pulpal floor was done for search of other orifices that were absent. On instrumentation, all scouting files converged into a single broad canal. Working length was determined using radiograph (Angle’s Method). Cleaning and shaping was done using step back technique using hand instrumentation. Irrigation between instrumentation was done using 5% of sodium hypochlorite solution (Hyposept, Asuwaldi, India). Final irrigation was done with normal saline (NS, Nirlife, India), and root canal space was obturated using cold lateral condensation of gutta percha and AH-Plus sealer (Dentsply-Maillefer, USA). The tooth was subsequently restored. On follow-up visits, the patient was clinically asymptomatic after 2 years (Figs. 1-3).

## Discussion

An adequate knowledge about root canal morphology and its variations forms the foundation to technically sound endodontic treatment (1). The variability of the root canal system of multirooted teeth is a formidable challenge to both diagnosis and treatment (2). Although such variations are rare, as far as the prognosis of individual cases is concerned, their importance is unequivocal. The location and morphology of root canals should be identified radiologically prior to the root canal treatment Root canal morphology should be examined further during treatment through the evaluation of radiographs taken from different horizontal angles. A good way to detect root canal morphology and anatomy is to take an initial preoperative radiograph and subsequently an additional radiograph from a 20-degree mesial or distal projection (3,4).

However, the drawbacks of radiographs have been well documented primarily due to it being a two-dimensional image of a three-dimensional object. The superimposition of multiple anatomic structures mainly the zygomatic bone and the overlapping of canals due to which the complexity of the canal system cannot be well appreciated often result in errors in proper diagnosis, hence questioning its reliability (5). Recent imaging tools like spiral CT and CBCT have emerged as valuable tools in the field of endodontics due to its accuracy, reliability and three-dimensional imaging capabilities. In CBCT, a cone-shaped beam rotates 360° around the patient to obtain a volume and hence it I possible to obtain a 3D reconstruction of the structures in question (6). Its uses in endodontics include identifying dental anatomic variations such as additional roots and/or canals, fused roots, identification of horizontal/vertical fracture line in the tooth root and management of internal and external resorptive defects (7). Also overzealous removal of tooth structure during access cavity preparation and exploration of root canal orificescan be avoided using CBCT. Tachibana and Matsumoto (1990) studied the applicability of CT to endodontics. They concluded that this method allowed the observation of the morphology of the root canals, the roots and the appearance of the tooth in every direction. Moreover, the image could be analyzed, altered and reconstructed by the computer (8). The disadvantages of a CT scan include the costs, availability and the higher ionizing radiation exposure. But recent studies have shown that low dose protocols are also available (9).

The maxillary first molar is considered as possibly the most treated, least understood, posterior tooth (10). A thorough knowledge of the root canal system is an absolute necessity for a successful root canal treatment. Weine divided the position of one or two canals within one root into four categories (Weine I–IV) (11). Vertucci described a classification encompassing eight different types (12).

There has been significant literature establishing the variations in the number of root canals of maxillary 1st molars. 5, 6 and 7 root canals in a single maxillary 1st molar have been recorded previously (13). The presence of the MB2 canal in a significant number of cases has established the fact that extra canals are more of a rule rather than an exception. However the clinician should also be wary of the fact that in a few cases, there is a possibility of fused or fewer canals. Otherwise the clinician’s efforts to locate additional canals may lead to iatrogenic errors such as perforations and furcation involvement (14). Conventional intra-oral periapical radiographs are an important diagnostic tool in endodontics for assessing the canal configuration.

This case report is one such case wherein we suspected missed canals initially but ended up finding only one single canal. In such doubtful cases, a radiograph cannot be considered to be foolproof because of its inherent limitations. Recently, newer diagnostic methods such as CT and SCT overcome the disadvantages of radiographs by providing a 3D image. These imaging techniques have emerged as a powerful tool for evaluation of root canal morphology. Thus, in this particular case, SCT was carried out to rule out any possibility of missed canals. Detailed analysis of the 3D reconstruction image at the cervical, middle and apical regions of the root excludes the possibility of multiple fused roots due to the lack of external root eminences on the buccal aspect. Moreover, there is absolutely no indication of any pulp canal space other than the one that is well centered within the confines of the root. Thus, it is safe to assume that the tooth in question is definitely maxillary first molar with single root and canal.

## Conclusions

Anatomical variations continue to be the most challenging aspect of achieving successful endodontic therapy. Hence it is no coincidence that more importance is given to extra canals, merging and demerging canals, apical deltas, and lateral canals but it is imperative that the clinician should also focus on the possibility of fewer canals.

To conclude, the present case highlights the need to develop meticulous observation skills on the part of the clinician to identify any aberrations from the normal within the tooth, possibly with the use of advanced imaging techniques available today.

This paper also highlights the role a SCT/CBCT as an objective analytical tool to ascertain root canal morphology.
